# Unveiling the benefits of Vitamin D3 with SGLT-2 inhibitors for hypertensive obese obstructive sleep apnea patients

**DOI:** 10.1186/s12967-025-06312-w

**Published:** 2025-03-07

**Authors:** Huai Heng Loh, Siow Phing Tay, Ai Jiun Koa, Mei Ching Yong, Asri Said, Chee Shee Chai, Natasya Marliana Abdul Malik, Anselm Ting Su, Bonnie Bao Chee Tang, Florence Hui Sieng Tan, Norlela Sukor

**Affiliations:** 1 Department of Medicine, Faculty of Medicine, Universiti Kebangsaan Malaysia, 56000 Kuala Lumpur, Malaysia; 2 Faculty of Medicine and Health Sciences, Universiti Malaysia Sarawak, 93350 Kota Samarahan, Sarawak Malaysia; 3 Department of Medicine, Sarawak General Hospital, 93586 Kuching, Sarawak Malaysia; 4 Hospital Canselor Tuanku Muhriz, 56000 Kuala Lumpur, Malaysia

**Keywords:** Cardiovascular, Dapagliflozin, Endothelial function, Epworth Sleepiness Scale, Heart rate variability

## Abstract

**Background:**

Obstructive sleep apnea (OSA) is associated with poorer quality of life (QoL) and increased cardiovascular risks, which may be exacerbated by hypovitaminosis D. Sodium glucose transporter-2 inhibitor (SGLT2i) provides cardiovascular benefits beyond glycemic control. As vitamin D3 and SGLT2i act through different pathways with similar mechanisms in improving cardio-metabolic health, this study aimed to investigate the synergistic effects of this combination therapy in improving these parameters and QoL in hypertensive obese OSA patients.

**Methods:**

Patients who fulfilled the study criteria were randomized to receive: (i) Dapagliflozin, (ii) vitamin D3, (iii) Dapagliflozin plus vitamin D3, or (iv) no treatment, for 16 weeks. The parameters evaluated included anthropometric measurements, uric acid, HbA1c, lipid profile, steatotic liver disease grade, plasma aldosterone concentration, plasma renin concentration, ultrasound flow-mediated dilatation of brachial artery, hsCRP, heart rate variability (HRV), Epworth Sleepiness Scale (ESS), and QoL scores.

**Results:**

A total of 163 patients were recruited and 153 completed the study. The combination of vitamin D3 and Dapagliflozin treatment led to significant improvements in metabolic parameters and nocturnal heart rates, and prevented deterioration of HRV, with healthier HRV at the end of study visit compared to the control group. Only the combination group exhibited improvements in both ESS and QoL scores.

**Conclusions:**

This is the first study to demonstrate beneficial effects of combining vitamin D3 and SGLT2i in cardio-metabolic outcomes and QoL in hypertensive obese OSA patients. These findings highlight the potential of this combination therapy in addressing the cardio-metabolic challenges and QoL in this patient population.

**Trials registration:**

NCT06690723. Registered 10 October 2024—Retrospectively registered, https://register.clinicaltrials.gov/prs/beta/studies/S000EWGF00000085/recordSummary

**Supplementary Information:**

The online version contains supplementary material available at 10.1186/s12967-025-06312-w.

## Background

Obstructive sleep apnea (OSA) is one of the most common sleep-related breathing disorders, with increasing prevalence worldwide due to its close association with obesity and aging [1]. Although still unclear, the recurrent episodes of upper airway obstruction during sleep leading to intermittent hypoxia and sleep fragmentation may contribute to endothelial dysfunction, cardiac autonomic dysfunction, and inflammatory processes [2]. This may explain the elevated cardiovascular risks observed in this cohort of patients. Furthermore, the presence of metabolic syndrome commonly found in OSA, especially hypertension, which has been reported to be highly prevalent in these patients, may worsen the cardio-metabolic risks [3]. Untreated OSA patients may also exhibit excessive daytime sleepiness [4] and report poorer quality of life (QoL) when compared to the general population [5]. The current gold standard treatment for OSA is continuous positive airway pressure (CPAP) treatment, however compliance has been reported to be poor and it does not confer metabolic improvement and cardiovascular protection [6, 7].

OSA is associated with activation of the renin–angiotensin–aldosterone system (RAAS), which partly contributes to the high prevalence of hypertension and plays a critical role in the pathophysiology of various cardiovascular disease and all-cause mortality seen in these patients [8–10]. Vitamin D, a fat-soluble secosteroid, is primarily obtained through sunlight exposure. Despite Malaysia’s abundant sunshine, vitamin D deficiency (VDD) remains prevalent [11]. Increasing evidence suggests a bidirectional relationship and independent correlation between OSA and VDD [12, 13]. This association remained significant even when age and body mass index (BMI) were controlled for, supporting the independent correlation between these two entities [13]. Beyond its effect on the skeletal system, vitamin D is now believed to play a role in cardiovascular health. Vitamin D receptors (VDR) and the enzymes responsible for metabolizing vitamin D are found in the cardiovascular tissues [14]. The mechanism of which vitamin D supplementation contributes to cardiovascular benefits is hypothesized to be driven by its effect on reducing inflammatory markers thus improving endothelial function [15], mitigating insulin resistance [16], and ameliorating RAAS derangement [17]. Although these benefits remain controversial, the observed inconsistencies may be attributed to heterogenous study designs, diverse populations, varying vitamin D dosages, and treatment duration differences [18]. Nevertheless, vitamin D supplementation has shown therapeutic benefits in addressing metabolic derangements, including improving glucose homeostasis [19], enhanced lipid profiles [20], and reduced inflammation [21], which could contribute to mitigating cardiovascular risks. However, data on vitamin D supplementation in patients with OSA remains limited.

The sodium glucose transporter-2 inhibitor (SGLT2i) is a class of oral anti-diabetic agent that provides cardiovascular benefits extending beyond its glucose-lowering effects. It works by inducing glycosuria and is known to reduce HbA1c and blood pressure (BP) in addition to increasing HDL and LDL while decreasing triglycerides [22]. Large cardiovascular trials such as DECLARE-TIMI and EMPA-REG have consistently shown a reduction in adverse cardiovascular outcomes with SGLT2i use [23, 24]. In addition to the metabolic improvements mentioned, the cardiovascular benefit stemming from the use of SGLT2i is also believed to be contributed by other mechanisms, such as direct cardiac beneficial effects including improvement in cardiac metabolism [25], reducing inflammatory markers [26], and natriuresis effect leading to reduction in BP [27]. Nevertheless, despite the favourable cardiovascular outcomes with the use of SGLT2i among patients with type 2 diabetes, data on the use of this drug among patients with OSA is scarce.

Since vitamin D and SGLT2i act through different pathways, with some similarities in their underlying mechanisms for improving cardiovascular and metabolic health, this study aimed to investigate the combined effects of these two treatments on (i) cardio-metabolic parameters, (ii) sleepiness symptoms, and (iii) QoL in hypertensive obese OSA patients .

## Materials and methods

### Study population and study criteria

This was a 4-arm parallel randomized controlled trial conducted from June 2022 till October 2024. Patients who were suspected of having OSA were referred from health clinics around Kuching and Kota Samarahan areas to Sarawak General Hospital Sleep Unit.

A lower BMI cut-off was used in this study as Asians have higher cardiovascular risks at a lower BMI than the existing World Health Organization (WHO) cut-off point for obesity [28]. Thus, the inclusion criteria were (i) aged ≥ 18 years, (ii) BMI ≥ 27.5 kg/m^2^, (iii) confirmed OSA on sleep study, and (iv) presence of hypertension; whereas the exclusion criteria were (i) known secondary hypertension, (ii) conditions that affect RAAS and vitamin D levels, including chronic kidney disease, congestive heart failure, recent myocardial infarction within 6 weeks of recruitment, and uncontrolled thyroid or parathyroid disorders, (iii) long-standing atrial fibrillation, (iv) CPAP treatment, (v) on vitamin D or calcium supplements, (vi) on SGLT2i treatment, (vii) on medications that could lead to weight loss, such as GLP-1 receptor analogue, (viii) malignancy, and (ix) pregnancy.

### Measurements and definitions

OSA was confirmed with sleep study, and its severity was classified as mild (apnoea hypopnea index, AHI 5–15/hour), moderate (AHI > 15–30/hour), or severe (AHI > 30/hour) in accordance to the clinical practice guidelines [29]. Hypertension was defined as systolic BP ≥ 140 mmHg, and/or diastolic BP ≥ 90 mmHg on two separate occasions, or if the patient was taking anti-hypertensive treatment. Previous smokers and previous alcohol consumers were those who ceased smoking or alcohol intake for at least 1 year respectively. Vitamin D status was categorized according to the Endocrine Society guidelines [30]. Those with serum 25-hydroxyvitamin D [25(OH)D] of < 20 ng/mL were considered vitamin D deficient, 25(OH)D 20–30 ng/mL as insufficient, whereas a level of > 30 ng/mL were sufficient.

Metabolic parameters assessed included BP, BMI, waist circumference, neck circumference, uric acid, HbA1c, lipid profile, and presence of metabolic-associated steatotic liver disease (MASLD); whereas cardiovascular risk parameters evaluated were (i) high-sensitivity C-reactive protein (hsCRP) reflecting inflammation, (ii) cardiac autonomic function using long-term heart rate variability (HRV), i.e. standard deviation of the NN interval (SDNN), mean of the 5 min SDNN calculated over 24 h (ASDNN), and the standard deviation of the average NN interval calculated over 5 min (SDANN), and short-term HRV using the Polar H10 device, which reported SDNN, mean RR, square root of the mean of the sum of the squares of differences between adjacent NN intervals (RMSSD), parasympathetic nervous system (PNS) index, sympathetic nervous system (SNS) index, and stress index, (iii) minimum, maximum, and average heart rates evaluated over 24 h, (iv) daytime and nocturnal heart rates, (v) RAAS components, including plasma aldosterone concentration (PAC) and plasma renin concentration (PRC), and (vi) endothelial function assessed with ultrasound of flow-mediated dilatation (FMD) of the brachial artery.

MASLD was graded as normal (grade 0) or grades 1–3[31]. The hsCRP levels were classified into < 1 mg/L, 1–3 mg/L, and > 3 mg/L as low-, intermediate-, and high-risk for global cardiovascular disease respectively[32]. The normal range of each HRV parameter is displayed in Supp Tables 1 and 2. Briefly, a higher SDNN, ASDNN, SDANN, mean RR, RMSSD and PNS index, with lower SNS index, stress index, and heart rates are indicative of a healthier heart, reflecting better autonomic regulation and cardiovascular resilience. The dipping percentage was defined using the following equation:

$${\text{Dipping percentage }}\left( \% \right)\; = \;\frac{{{\text{Daytime heart rate }}{-}{\text{ Nocturnal heart rate}}}}{{\text{Daytime heart rate}}} \times 100$$

A change of < 10% was considered as non-dipping. Daytime and nocturnal episodes were defined as occurring from 11am to 10 pm, and from 12 midnight to 4 am, respectively.

The normal ranges of PAC and PRC were 2.21–35.3 ng/dL and 4.4–46.1 μIU/mL respectively for samples taken in upright posture.

The FMD value was calculated using the following equation and expressed as a percent change in vessel caliber:

$${\text{FMD }}\left( \% \right)\; = \,\frac{{\left( {{\text{Peak Diameter }}{-}{\text{ Baseline Diameter}}} \right)}}{{\text{Baseline Diameter}}} \times {1}00$$

Normal endothelial function assessed by FMD was defined as ≥ 7.1% as this cut-off best discriminates those with cardiovascular risks compared to those without [33].

Sleepiness symptoms were evaluated using Epworth Sleepiness Scale (ESS), a self-administered questionnaire with scores ranging from 0 to 24, of which a score of > 10 indicates higher than normal daytime sleepiness.

QoL was evaluated using the WHOQOL-BREF, which is a shorter version of WHOQOL-100 [34]. It consists of 26 self-administered questions regarding an individual’s perception on health and well-being over the previous two weeks, with responses on a 1–5 Likert scale. The four domains covered by WHOQOL-BREF include (i) physical health, (ii) psychological health, (iii) social relationships, and (iv) environmental health, in addition to two separate questions asking specifically about the individual’s overall perception on overall quality of life and general health.

### Study protocol (Supp Fig)

All anti-hypertensive treatments that affect the RAAS were discontinued for at least 6 weeks for spironolactone, 4 weeks for non-potassium sparing diuretics, and 2 weeks for angiotensin converting enzyme inhibitors, angiotensin II type 1 receptor blockers, beta blockers, and dihydropyridine calcium blockers before first visit (V1). If indicated, anti-hypertensive treatment with the least effect on RAAS, such as non-dihydropyridine calcium blockers and/or alpha blockers, were prescribed for BP control. All participants had unrestricted salt intake.

After informed consent, the study subjects were interviewed by a single investigator regarding their socio-demography and medical history. Following administration of ESS and WHOQoL questionnaires, all subjects then underwent anthropometric measurements by the same investigator. Blood was taken after an overnight fast for at least 8 h and patients being upright for at least 2 h upon waking.

Ultrasound of the hepatobiliary system and FMD of the brachial artery were then performed by a single radiologist to assess for presence of steatotic liver disease and endothelial dysfunction respectively. Subsequently, all patients underwent evaluation of the cardiac autonomic function using Polar H10 device to assess short-term HRV, followed by placement of a 24 h Holter monitoring to evaluate the long-term HRV, while maintaining their usual daily activities.

The subjects were then randomized by the primary investigator using computer-generated block randomization method to either of the 4 arms: (i) Group 1: Dapagliflozin, (ii) Group 2: vitamin D3, (iii) Group 3: Dapagliflozin plus vitamin D3, or (iv) Group 4 (control arm): no treatment, for a total duration of 16 weeks. All patients received standard education by a single investigator on lifestyle modification at V1. At the same setting, those who received Dapagliflozin were prescribed 10 mg daily and were given standard advice of precautions with its use [35]. Patients who received vitamin D3 had the doses of vitamin D prescribed according to the baseline 25(OH)D level. Those with 25(OH)D level of < 30 ng/mL received 6000 IU a day, otherwise they were prescribed with vitamin D3 dose of 4000 IU a day [30].

A follow-up phone call was made at 1-week and 4-week after V1 to inquire if the patients developed any side effects with the medications. All patients were seen 8 weeks later at visit 2 (V2) to document changes in metabolic parameters, endothelial function, and short-term HRV. The vitamin D3 dose was titrated according to the 25(OH)D level at V2, if indicated. All patients underwent the same evaluations as V1 at the end of the study (V3). Any side effects encountered during the study period were documented at each clinic visit. Compliance of the patients to treatment was assessed using pill-counting method. Those who had a compliance rate of < 80% were removed from the final analysis. All except the primary investigator were unaware of the patients’ study group.

Subgroup analysis was also performed to evaluate the effect of treatment on cardio-metabolic parameters among those who were vitamin D insufficient or deficient at baseline in all study arms, and those who achieved vitamin D sufficiency among those who received vitamin D treatment. Besides, gender-based analysis was performed to compare the effect of treatment combination on cardiovascular and metabolic parameters.

### Statistical analysis

Statistical analysis was performed using SPSS software (version 29, SPSS Inc., Chicago IL). Continuous variables were presented as mean ± SD or median (IQR), whereas categorical variables were presented as absolute counts with their percentages. Variables among the 4 groups were compared at baseline and end of study visit using ANOVA if they were normally distributed, and comparison between groups were performed using Tukey–Kramer post hoc test if the differences on ANOVA analysis were significant. Otherwise, the differences were compared using Kruskal–Wallis test. For categorical variables, chi-squared test was applied to test the differences among the 4 groups. For normally distributed variables, the changes with treatment were compared using T test for baseline and end of study visits, or repeated measures ANOVA for all three study visits. For non-normally distributed variables, the changes were evaluated using Wilcoxon signed rank test for comparison between 2 study visits, or Friedman test for 3 study visits. A p value of < 0.05 was taken as statistically significant.

## Results

A total of 797 patients suspected of having OSA were referred for sleep study over the period of 29 months. After screening of eligibility, 163 who fulfilled study criteria were recruited. A total of 10 patients were removed from analysis as 6 were found to be non-compliant to treatment, whereas 4 defaulted follow-up visits due to logistic reasons. Hence, 153 patients were included in the final analysis. There was no significant difference in the baseline demography, ESS, QoL scores, and cardio-metabolic parameters, as shown in Tables 1 and 2.

**Table 1:** Baseline demography

Variables	Group 1 n = 36	Group 2 n = 41	Group 3 n = 39	Group 4 n = 37	p
*Demography*					
Age, years	44.7 ± 12.3	48.1 ± 11.6	46.8 ± 11.6	45.1 ± 12.2	0.574
Male, n (%)	20 (55.6)	19 (46.3)	24 (61.5)	21 (56.8)	0.580
Ethnicity, n (%)					0.890
Malay	15 (41.7)	20 (48.8)	17 (43.6)	15 (40.5)	
Chinese	7 (19.4)	6 (14.6)	8 (20.5)	9 (24.3)	
Iban	7 (19.4)	5 (12.2)	7 (17.9)	4 (10.8)	
Bidayuh	6 (16.7)	10 (24.4)	5 (12.8)	7 (18.9)	
Others	1 (2.8)	0 (0)	2 (5.1)	2 (5.4)	
Education, n (%)					0.112
No Formal Education	1 (2.8)	1 (2.4)	1 (2.6)	1 (2.7)	
Primary	3 (8.3)	11 (26.8)	3 (7.7)	1 (2.7)	
Secondary	22 (61.1)	16 (39.0)	21 (53.8)	23 (62.2)	
Tertiary	10 (27.8)	13 (31.7)	14 (35.9)	12 (32.4)	
Co-morbidities, n (%)					
Type 2 diabetes	19 (52.8)	27 (65.9)	28 (71.8)	18 (48.6)	0.132
Dyslipidemia	24 (64.9)	24 (58.5)	25 (62.5)	23 (62.2)	0.952
Hyperuricemia	20 (55.6)	29 (70.7)	24 (61.5)	23 (62.2)	0.584
Smoking, n (%)					0.693
Yes	4 (11.1)	6 (15.0)	8 (21.6)	4 (10.8)	
No	22 (61.1)	21 (52.5)	19 (51.4)	25 (67.6)	
Previous	10 (27.8)	13 (32.5)	10 (27.0)	8 (21.6)	
Alcohol consumer, n (%)					0.461
Yes	7 (19.4)	6 (15.0)	8 (21.6)	11 (29.7)	
No	22 (61.1)	27 (67.5)	24 (64.9)	24 (64.9)	
Previous	7 (19.4)	7 (17.5)	5 (13.5)	2 (5.4)	
OSA severity, n (%)					0.977
Mild	3 (8.3)	4 (9.8)	2 (5.1)	2 (5.4)	
Moderate	10 (27.8)	12 (29.3)	10 (25.6)	11 (29.7)	
Severe	23 (63.9)	25 (61.0)	27 (69.2)	24 (64.9)	
AHI, /hour	45.3 ± 27.0	41.7 ± 24.0	50.2 ± 30.6	46.3 ± 28.2	0.593
*Anthropometry*					
Systolic BP, mmHg	153.3 ± 16.5	150.6 ± 17.1	151.2 ± 13.8	153.8 ± 21.2	0.811
Diastolic BP, mmHg	97.4 ± 11.7	94.1 ± 11.6	97.2 ± 13.0	98.2 ± 15.5	0.524
Pulse rate, bpm	76.5 ± 13.9	75.4 ± 13.3	76.4 ± 12.2	80.7 ± 13.8	0.319
BMI, kg/m^2^	42.1 ± 7.4	39.8 ± 7.3	41.6 ± 7.0	39.4 ± 7.8	0.328
Neck circumference, cm	43.5 ± 4.7	42.8 ± 3.9	44.4 ± 4.0	43.1 ± 4.3	0.357
Waist circumference, cm	122.5 ± 14.6	117.4 ± 12.0	122.3 ± 12.6	117.0 ± 15.9	0.147
*Patient-reported outcomes*					
ESS	8.8 ± 5.0	8.9 ± 5.8	10.4 ± 4.7	9.1 ± 5.7	0.585
WHOQoL-BREF					
Quality of Life	3.49 ± 0.77	3.39 ± 0.70	3.40 ± 0.81	3.61 ± 0.72	0.772
General Health	2.97 ± 0.87	3.02 ± 0.69	2.95 ± 0.64	3.11 ± 0.97	0.726
Domain 1	12.2 ± 1.7	12.5 ± 1.7	12.7 ± 1.6	12.8 ± 1.7	0.653
Domain 2	12.8 ± 1.9	12.5 ± 1.6	13.1 ± 2.1	13.1 ± 1.7	0.844
Domain 3	14.4 ± 2.5	13.9 ± 2.8	13.9 ± 2.9	14.5 ± 3.4	0.733
Domain 4	14.1 ± 2.2	13.7 ± 2.1	14.1 ± 1.9	14.6 ± 1.8	0.452

Numerical variables are presented as the mean ± standard deviation, categorical variables are defined as absolute count and percentage

OSA: obstructive sleep apnea; AHI: apnea hypopnea index; BP: blood pressure; BMI: body mass index; ESS: Epworth Sleepiness Scale; WHOQoL-BREF: World Health Organization Quality of Life—brief version

**Table 2:** Cardio-metabolic parameters of study participants at baseline

Variables	Group 1 n = 36	Group 2 n = 41	Group 3 n = 39	Group 4 n = 37	p
*Biochemistry*					
Uric acid, mmol/L	420.4 ± 84.9	427.2 ± 103.8	433.9 ± 87.3	422.7 ± 85.0	0.923
HbA1c, %	6.4 (5.9, 6.8)	6.3 (5.9, 6.9)	6.7 (5.9, 7.7)	6.0 (5.8, 6.8)	0.202
Lipid, mmol/L					
Total	4.61 ± 1.32	4.95 ± 0.87	4.52 ± 1.03	4.59 ± 0.94	0.339
LDL-C	2.65 ± 1.08	2.86 ± 0.79	2.67 ± 0.78	2.61 ± 0.81	0.593
HDL-C	1.27 ± 0.23	1.33 ± 0.26	1.29 ± 0.42	1.29 ± 0.26	0.872
Triglyceride	1.35 (1.08, 2.11)	1.65 (1.13, 2.33)	1.54 (1.12, 1.96)	1.65 (1.01, 2.16)	0.611
Creatinine, umol/L	72.4 ± 19.3	74.3 ± 25.7	74.4 ± 17.1	74.8 ± 22.2	0.966
25(OH)D, ng/mL	18.3 ± 7.6	19.6 ± 9.1	20.3 ± 5.7	21.0 ± 8.1	0.493
Vitamin D status, n (%)					0.274
Deficient	22 (59.5)	25 (61.0)	19 (48.7)	20 (55.6)	
Insufficient	12 (32.4)	10 (24.4)	19 (48.7)	13 (36.1)	
Sufficient	3 (8.1)	6 (14.6)	1 (2.6)	3 (8.3)	
Phosphate, mmol/L	1.14 ± 0.19	1.19 ± 0.24	1.18 ± 0.22	1.20 ± 0.15	0.664
iPTH, pg/mL	68.2 (43.8, 91.8)	65.7 (40.7, 88.8)	65.3 (50.5, 87.2)	56.1 (40.3, 72.8)	0.421
hsCRP	6.8 ± 9.9	6.3 ± 5.6	7.2 ± 5.6	8.4 ± 9.6	0.679
*Ultrasound of the hepatobiliary system*					
MASLD, n (%)					0.707
Normal	7 (19.4)	3 (7.3)	2 (5.1)	3 (8.1)	
Grade 1	6 (16.7)	10 (24.4)	8 (20.5)	6 (16.2)	
Grade 2	21 (58.3)	25 (61.0)	23 (59.0)	23 (62.2)	
Grade 3	2 (5.6)	3 (7.3)	5 (12.8)	4 (10.8)	
*Heart rate variability*					
Long-term, ms					
ASDNN	49.7 ± 21.4	48.7 ± 15.6	47.5 ± 14.8	44.4 ± 17.1	0.579
SDANN	105.5 ± 45.6	104.0 ± 39.2	96.5 ± 34.0	100.7 ± 27.6	0.725
SDNN	118.9 ± 48.4	116.9 ± 37.8	111.9 ± 37.6	111.9 ± 30.9	0.815
Short-term, ms					
SDNN, ms	17.9 (11.1, 25.0)	20.2 (13.4, 30.2)	16.8 (11.4, 28.0)	17.0 (11.8, 21.8)	0.647
Mean RR, ms	787.8 ± 150.0	794.4 ± 158.3	761.4 ± 148.0	753.7 ± 127.5	0.571
RMSSD, ms	16.5 (8.0, 25.3)	17.5 (10.5, 29.8)	15.0 (8.0, 22.0)	15.5 (10.0, 22.8)	0.657
PNS index	− 1.1 ± 1.2	− 1.1 ± 1.3	− 1.3 ± 1.1	− 1.5 ± 0.9	0.404
SNS index	3.0 ± 2.8	2.7 ± 3.1	3.3 ± 3.2	3.4 ± 3.0	0.694
Stress index	23.3 ± 12.1	21.4 ± 13.6	24.4 ± 15.3	23.4 ± 12.0	0.762
24 h heart rate, bpm					
Minimum	50.3 ± 8.9	49.1 ± 9.5	49.9 ± 9.5	52.8 ± 9.0	0.341
Maximum	123.3 ± 16.3	124.8 ± 13.7	122.7 ± 14.3	127.6 ± 13.7	0.470
Average	80.6 ± 11.1	80.8 ± 9.8	81.9 ± 9.3	84.1 ± 9.4	0.395
Daytime	84.2 ± 12.3	85.1 ± 9.9	83.1 ± 11.3	88.1 ± 10.7	0.426
Nocturnal	71.2 ± 13.0	71.9 ± 11.9	72.9 ± 10.3	74.4 ± 10.1	0.686
*Renin–angiotensin–aldosterone parameters*					
PAC, ng/dL	8.98 (5.48, 12.10)	9.36 (6.66, 11.4)	9.41 (6.59, 13.30)	8.82 (6.54, 11.30)	0.820
PRC, μIU/mL	13.03 (7.10, 23.67)	13.50 (6.88, 20.91)	13.68 (5.05, 23.94)	14.76 (7.45, 24.72)	0.949
*Endothelial function*					
FMD, %	6.17 ± 1.31	5.92 ± 1.80	6.12 ± 1.54	6.06 ± 1.21	0.890
FMD < 7%, n (%)	30 (83.3)	35 (85.4)	33 (84.6)	34 (91.9)	0.713

Numerical variables are presented as the mean ± standard deviation or median (IQR), categorical variables are defined as absolute count and percentage

25(OH)D: 25-hydroxyvitamin D; iPTH: intact parathyroid hormone; hsCRP: high-sensitivity C-reactive protein; MASLD: metabolic-associated steatotic liver disease; ASDNN: the mean of the 5-min SDNN calculated over 24 h; SDANN: the standard deviation of the average NN interval calculated over 5 min; SDNN: standard deviation of the NN interval; RMSSD: the square root of the mean of the sum of the squares of differences between adjacent NN intervals; PNS: parasympathetic nervous system; SNS: sympathetic nervous system; PAC: plasma aldosterone concentration; PRC: plasma renin concentration; FMD: flow-mediated dilatation

### Effect of treatment on metabolic parameters

The effect of treatment on metabolic parameters is presented in Table 3. All patients except those in the control arm demonstrated significant improvement in metabolic parameters. Specifically, patients in groups 1 and 3 demonstrated significant improvement in BP, BMI, neck and waist circumferences, uric acid, and HbA1c. A reduction in HDL-C level was noted in both groups. Patients in group 2 demonstrated significant improvement in systolic BP, uric acid, and total cholesterol levels. Two patients in group 1 had improvement of MASLD – one from grade 1 to normal, and another from grade 2 to normal; whereas 2 patients from group 3 showed improvement of MASLD from grade 1 to normal, although these changes did not achieve statistical significance. These findings were similar in the subgroup analysis (Supp Table 3). When comparing the improvements observed in group 3 by gender, females demonstrated more substantial enhancements in metabolic parameters than males (Supp Table 4).

**Table 3:** Metabolic parameter changes before and after treatment (all cohort)

Variables	Group 1 n = 36			Group 2 n = 41			Group 3 n = 39			Group 4 n = 37		
	Before	After	p	Before	After	p	Before	After	p	Before	After	p
*Anthropometric*												
Systolic BP, mmHg	153.3 ± 16.5	146.2 ± 15.3	0.030	150.6 ± 17.1	142.9 ± 16.0	0.003	151.2 ± 13.8	143.7 ± 16.5	0.003	153.8 ± 21.2	149.3 ± 20.6	0.098
Diastolic BP, mmHg	97.4 ± 11.7	93.7 ± 12.4	0.070	94.1 ± 11.6	91.0 ± 11.0	0.050	97.2 ± 13.0	92.8 ± 11.2	0.017	98.2 ± 15.5	96.4 ± 13.9	0.351
Pulse rate, bpm	76.5 ± 13.9	74.8 ± 14.1	0.197	75.4 ± 13.3	72.2 ± 13.4	0.096	76.4 ± 12.2	75.2 ± 11.1	0.527	80.7 ± 13.8	77.0 ± 14.1	0.057
BMI, kg/m^2^	42.1 ± 7.4	40.8 ± 6.5	< 0.001	39.8 ± 7.3	39.9 ± 7.5	0.646	41.6 ± 7.0	40.3 ± 6.5	< 0.001	39.4 ± 7.8	39.4 ± 8.0	0.649
Neck circ., cm	43.5 ± 4.7	42.5 ± 5.0	< 0.001	42.8 ± 3.9	42.6 ± 3.9	0.275	44.4 ± 4.0	43.4 ± 4.1	< 0.001	43.1 ± 4.3	42.9 ± 4.4	0.352
Waist circ., cm	122.5 ± 14.6	118.5 ± 13.2	< 0.001	117.7 ± 12.1	116.9 ± 12.6	0.190	122.3 ± 12.6	118.2 ± 12.2	< 0.001	117.0 ± 15.9	116.4 ± 16.2	0.316
*Biochemistry*												
Uric acid, mmol/L	420.4 ± 84.9	346.7 ± 89.5	< 0.001	427.2 ± 103.8	402.1 ± 92.7	0.036	433.9 ± 87.3	362.5 ± 86.5	< 0.001	422.7 ± 85.0	419.6 ± 83.7	0.744
HbA1c, %	6.4 (5.9, 6.8)	6.1 (5.7, 6.6)	0.005	6.3 (5.9, 7.0)	6.2 (5.8, 7.1)	0.773	6.7 (5.9, 7.7)	6.0 (5.7, 6.9)	< 0.001	6.0 (5.8, 6.8)	6.1 (5.7, 6.7)	0.387
Lipid, mmol/L												
Total	4.61 ± 1.32	4.32 ± 1.04	0.029	4.95 ± 0.87	4.68 ± 1.13	0.023	4.62 ± 1.03	4.33 ± 1.01	0.056	4.59 ± 0.94	4.59 ± 0.94	0.974
LDL-C	2.65 ± 1.08	2.52 ± 0.92	0.235	2.86 ± 0.79	2.74 ± 0.94	0.262	2.67 ± 0.78	2.51 ± 0.76	0.149	2.61 ± 0.81	2.69 ± 0.85	0.547
HDL-C	1.27 ± 0.23	1.17 ± 0.21	< 0.001	1.33 ± 0.26	1.27 ± 0.29	0.109	1.29 ± 0.42	1.14 ± 0.23	0.029	1.29 ± 0.26	1.22 ± 0.21	0.095
Triglyceride	1.35 (1.08, 2.11)	1.29 (1.05, 2.16)	0.236	1.65 (1.13, 2.33)	1.61 (1.12, 1.96)	0.095	1.54 (1.12, 1.96)	1.47 (1.02, 1.99)	0.042	1.65 (1.01, 2.16)	1.68 (1.24, 2.24)	0.507
*Ultrasound of hepatobiliary system*												
MASLD, n (%)			0.50			1.0			0.50			1.0
Normal	7 (18.4)	9 (23.7)		3 (7.1)	3 (7.1)		2 (5.4)	4 (10.8)		3 (8.3)	3 (8.3)	
Grade 1	6 (15.8)	5 (13.2)		10 (23.8)	10 (23.8)		8 (21.6)	6 (16.2)		6 (16.7)	6 (16.7)	
Grade 2	23 (60.5)	22 (57.9)		26 (61.9)	26 (61.9)		21 (56.8)	21 (56.8)		23 (63.9)	23 (63.9)	
Grade 3	2 (5.3)	2 (5.3)		3 (7.1)	3 (7.1)		6 (16.2)	6 (16.2)		4 (11.1)	4 (11.1)	

Numerical variables are presented as the mean ± standard deviation or median (IQR), categorical variables are defined as absolute count and percentage

BP: blood pressure; BMI: body mass index; circ: circumference; MASLD: metabolic-associated steatotic liver disease

### Effect of treatment on cardiovascular parameters

The effect of treatment on cardiovascular parameters in all study subjects is presented in Table 4. Only group 3 demonstrated significant improvement in the 24 h average and nocturnal heart rates. Groups 1 and 4 showed deterioration of the short-term HRV parameters over 16 weeks. Although there was significant improvement of FMD in all groups, only groups 2 and 3 had significant increment of the number of patients who achieved normal FMD at the end of study visits.

**Table 4:** Cardiovascular parameter changes before and after treatment in all cohort

Variables	Group 1 n = 36			Group 2 n = 41			Group 3 n = 39			Group 4 n = 37		
	Before	After	p	Before	After	p	Before	After	p	Before	After	p
hsCRP	6.8 ± 9.9	6.5 ± 5.8	0.903	6.3 ± 5.6	5.5 ± 5.0	0.050	7.2 ± 5.6	7.6 ± 5.3	0.587	8.4 ± 9.6	6.5 ± 6.5	0.017
*Long term HRV, ms*												
ASDNN	49.7 ± 21.4	52.9 ± 21.2	0.228	48.7 ± 15.6	53.0 ± 16.1	0.053	47.5 ± 14.8	48.6 ± 15.6	0.700	44.4 ± 17.1	44.3 ± 17.3	0.946
SDANN	105.5 ± 45.6	114.1 ± 39.6	0.065	104.0 ± 39.2	109.2 ± 44.3	0.258	96.5 ± 34.0	102.5 ± 33.3	0.078	100.7 ± 27.6	102.1 ± 31.2	0.750
SDNN	118.9 ± 48.4	128.6 ± 42.5	0.072	116.9 ± 37.8	123.2 ± 40.4	0.194	111.9 ± 37.6	115.9 ± 32.3	0.387	111.9 ± 30.9	114.1 ± 33.9	0.637
*Short term HRV*												
SDNN, ms	17.9 (11.1, 25.0)	12.5 (7.8, 21.8)	0.026	20.2 (13.4, 30.2)	15.2 (8.8, 28.2)	0.208	16.8 (11.6, 28.5)	16.4 (10.1, 31.0)	0.694	17.0 (11.8, 21.8)	11.0 (8.5, 18.8)	0.005
Mean RR, ms	787.8 ± 145.0	748.8 ± 147.5	0.010	786.7 ± 155.4	777.6 ± 133.6	0.620	761.4 ± 149.0	786.8 ± 147.0	0.146	753.7 ± 127.5	735.1 ± 134.3	0.238
RMSSD, ms	16.5 (8.0, 25.3)	10.5 (6.0, 19.0)	0.032	17.5 (10.5, 29.8)	16.0 (7.5, 29.0)	0.142	15.5 (9.5, 22.0)	13.0 (9.0, 20.0)	0.803	15.5 (10.0, 22.8)	10.0 (7.0, 14.8)	0.007
PNS index	− 1.3 ± 1.0	− 1.7 ± 0.9	0.003	− 1.1 ± 1.3	− 1.2 ± 1.1	0.555	− 1.3 ± 1.1	− 1.3 ± 0.9	0.935	− 1.5 ± 0.9	− 1.7 ± 0.9	0.051
SNS index	3.0 ± 2.8	4.0 ± 3.5	0.006	2.8 ± 3.2	3.0 ± 2.6	0.555	3.3 ± 3.2	3.1 ± 2.3	0.527	3.4 ± 3.0	4.4 ± 3.1	0.019
Stress index	22.1 ± 10.0	26.4 ± 14.3	0.016	21.7 ± 13.9	23.9 ± 12.6	0.390	24.4 ± 15.3	23.9 ± 10.2	0.817	23.4 ± 12.0	27.8 ± 10.4	0.035
*24 h heart rate, bpm*												
Min HR	50.3 ± 8.9	48.7 ± 7.9	0.217	49.1 ± 9.5	47.8 ± 8.4	0.282	49.9 ± 9.5	49.4 ± 6.8	0.643	52.8 ± 9.0	51.6 ± 8.2	0.223
Max HR	123.3 ± 16.3	122.1 ± 16.3	0.592	124.8 ± 13.7	122.5 ± 13.2	0.206	122.7 ± 14.3	121.7 ± 14.8	0.630	127.6 ± 13.7	125.2 ± 13.1	0.110
Avg HR	80.6 ± 11.1	78.6 ± 10.5	0.085	80.8 ± 9.8	78.7 ± 9.6	0.114	81.9 ± 9.3	79.4 ± 9.5	0.008	84.1 ± 9.4	83.9 ± 9.8	0.866
Day HR	84.2 ± 12.3	84.0 ± 11.7	0.874	85.1 ± 9.9	83.1 ± 11.3	0.182	85.6 ± 9.2	84.2 ± 9.8	0.232	88.1 ± 10.7	88.6 ± 11.2	0.735
Noct. HR	71.2 ± 13.0	69.0 ± 10.7	0.141	71.9 ± 11.9	69.3 ± 11.0	0.103	72.9 ± 10.3	70.0 ± 9.0	0.003	74.4 ± 10.1	74.5 ± 10.5	0.943
Dipping, %	15.3 ± 11.1	17.5 ± 9.6	0.148	15.6 ± 9.3	16.2 ± 10.6	0.678	14.8 ± 6.9	16.5 ± 8.0	0.189	15.4 ± 7.4	15.6 ± 9.0	0.890
Non-dipper, n (%)	9 (24.3)	6 (16.2)	0.375	11 (27.5)	9 (22.5)	0.754	9 (23.1)	7 (18.4)	0.754	10 (27.8)	9 (25.0)	1.0
*Renin–angiotensin–aldosterone system parameters*												
PAC, ng/dL	8.96 (5.48, 12.10)	10.45 (7.58, 12.78)	0.113	9.36 (6.66, 11.40)	7.62 (4.96, 10.85)	0.464	10.10 (6.83, 13.55)	9.78 (6.70, 12.93)	0.252	8.82 (6.54, 11.30)	7.89 (6.76, 11.15)	0.934
PRC, μIU/mL	13.03 (7.10, 23.67)	15.50 (9.75, 35.31)	0.523	13.50 (6.88, 20.91)	13.30 (5.67, 26.42)	0.979	15.27 (6.02, 23.32)	12.77 (5.05, 29.20)	0.492	14.76 (7.45, 24.72)	14.67 (8.16, 23.91)	0.789
*Endothelial function*												
FMD, %	6.17 ± 1.31	6.70 ± 1.04	< 0.001	5.92 ± 1.80	6.61 ± 1.71	0.004	6.12 ± 1.50	6.60 ± 1.25	0.010	6.06 ± 1.21	6.51 ± 0.83	0.001
> 7.1%	6 (16.7)	11 (30.6)	0.125	6 (14.6)	12 (29.3)	0.031	6 (15.4)	13 (33.3)	0.016	3 (8.1)	7 (18.9)	0.125

Numerical variables are presented as the mean ± standard deviation or median (IQR), categorical variables are defined as absolute count and percentage

hsCRP: high-sensitivity C-reactive protein; ASDNN: the mean of the 5 min SDNN calculated over 24 h; SDANN: the standard deviation of the average NN interval calculated over 5 min; SDNN: standard deviation of the NN interval; RMSSD: the square root of the mean of the sum of the squares of differences between adjacent NN intervals; PNS: parasympathetic nervous system; SNS: sympathetic nervous system; min: minimum; max: maximum; avg: average; noct: nocturnal; PAC: plasma aldosterone concentration; PRC: plasma renin concentration; FMD: flow mediated dilatation; 25(OH)D: 25-hydroxyvitamin D

**Fig. 1:**
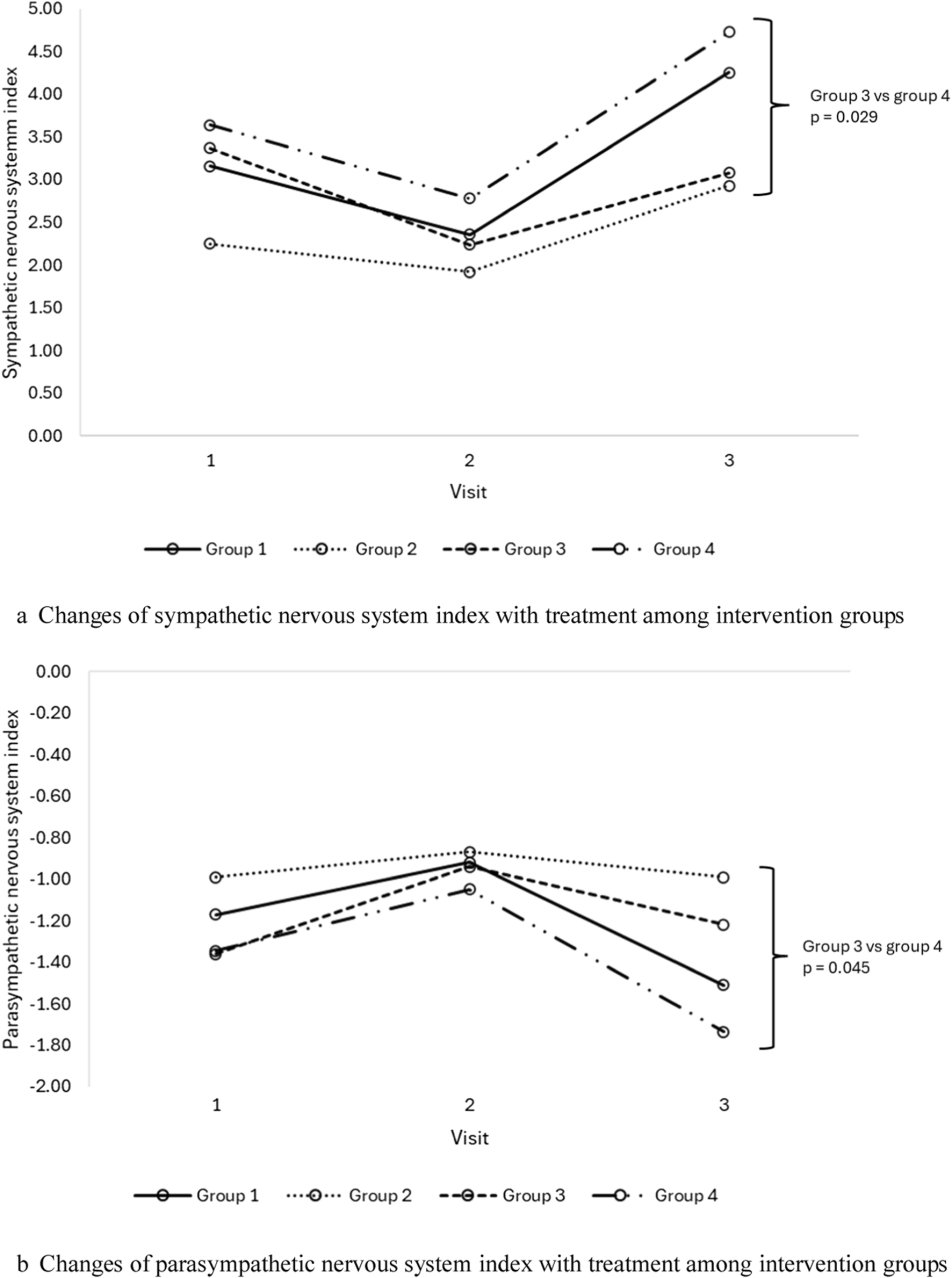

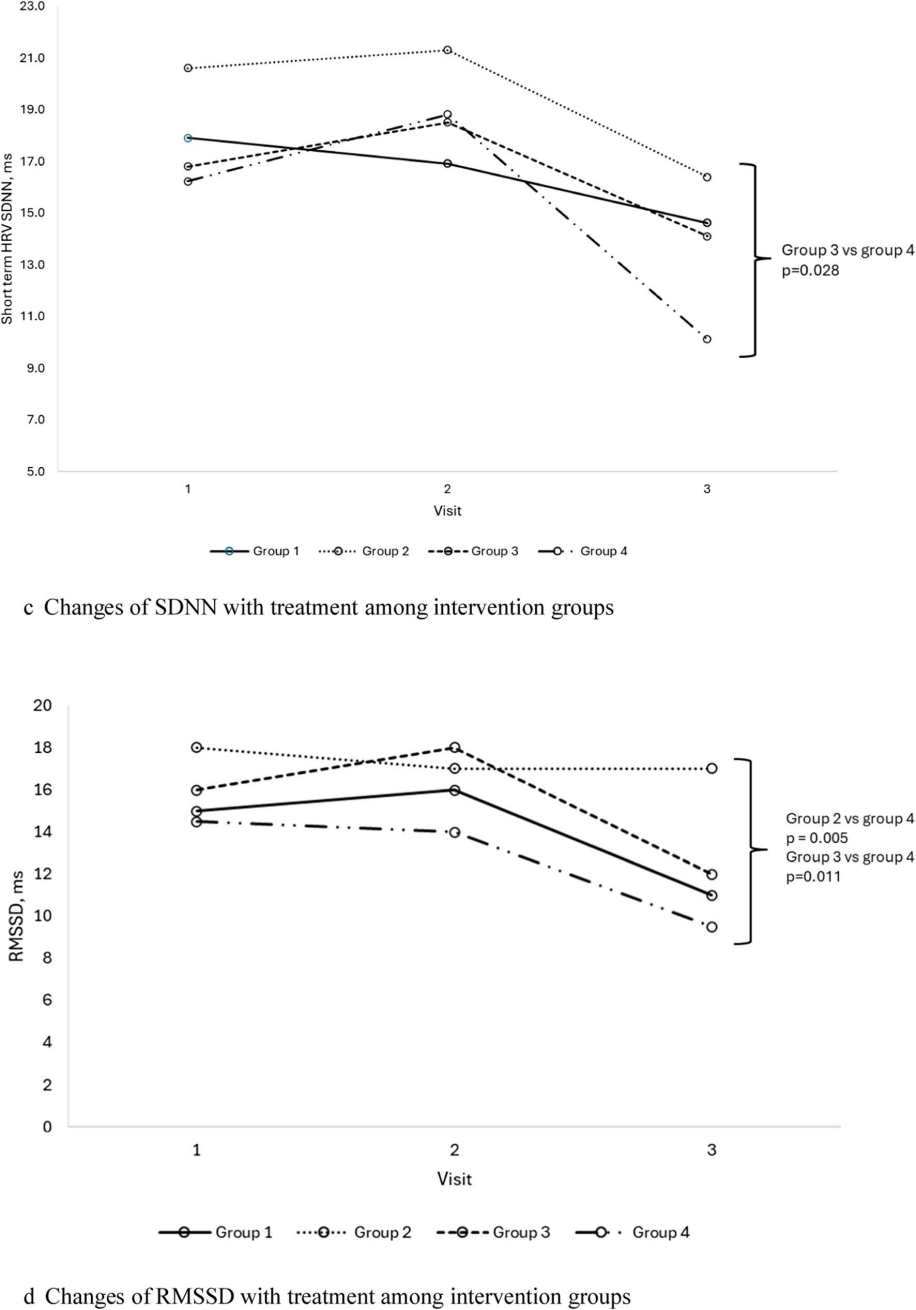

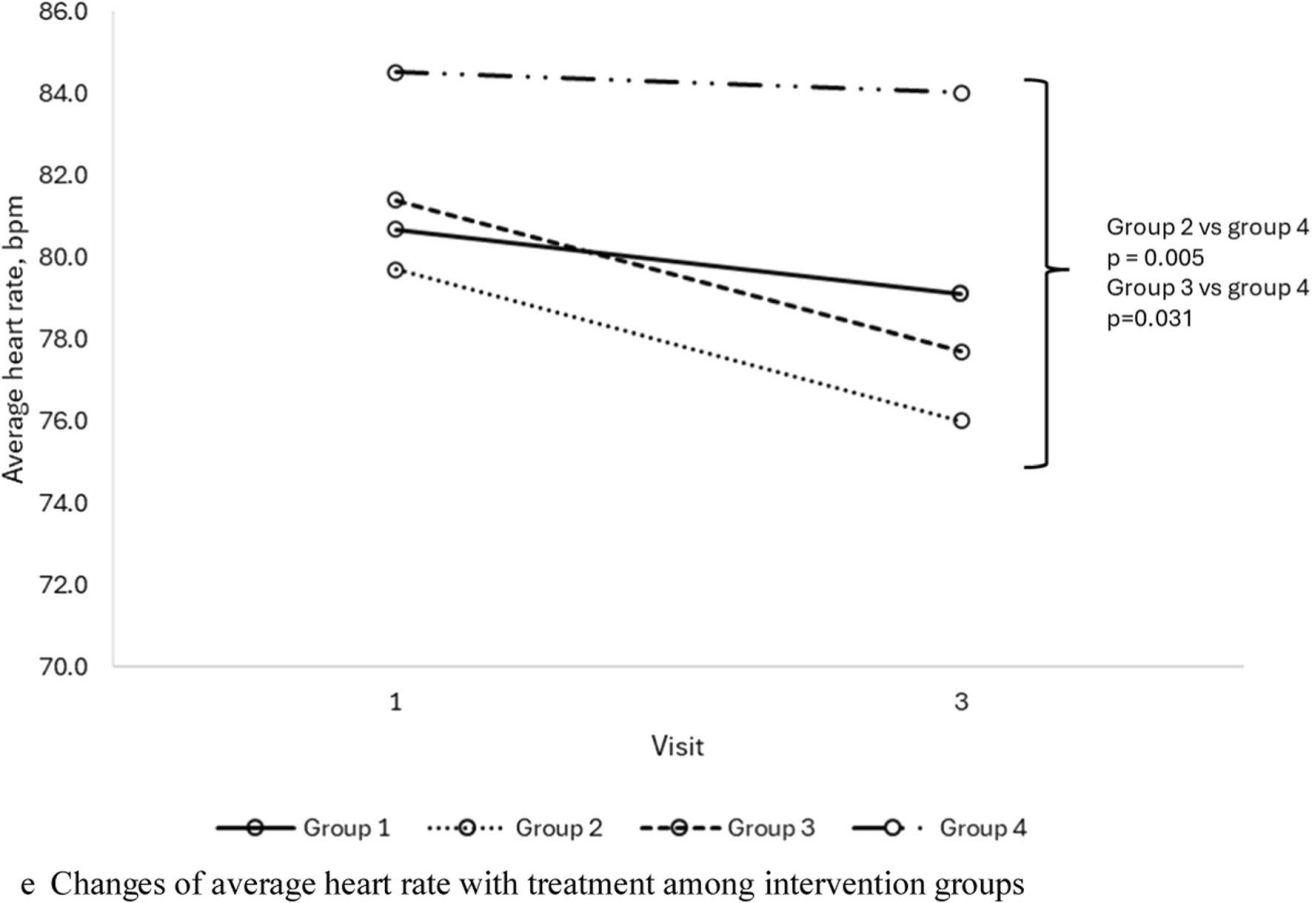
**a** Changes of sympathetic nervous system index with treatment among intervention groups. **b** Changes of parasympathetic nervous system index with treatment among intervention groups. **c** Changes of SDNN with treatment among intervention groups. **d** Changes of RMSSD with treatment among intervention groups. **e** Changes of average heart rate with treatment among intervention groups

Subgroup analysis demonstrated significant improvement in ASDNN and average heart rate in group 2; and significant improvement in mean RR and 24 h average and nocturnal heart rates in group 3 (Table 5). There were also significant differences noted in SNS index, PNS index, SDNN, RMSSD, and average heart rate at the end of study visit between groups 2 and 3 with group 4 (Fig. 1). Specifically, group 3 demonstrated significantly lower SNS index, and higher PNS index and SDNN, whilst groups 2 and 3 showed significantly higher RMSSD and lower average heart rate, at the end of study visit compared to group 4. Gender-based analysis of group 3 revealed that females exhibited more pronounced cardiovascular improvements compared to males (Supp Table 4).

**Table 5:** Cardiovascular parameter changes with treatment in subgroup analysis*

Variables	Group 1 n = 33			Group 2 n = 29			Group 3 n = 32			Group 4 n = 33		
	Before	After	p	Before	After	p	Before	After	p	Before	After	p
hsCRP	6.8 ± 10.1	6.9 ± 5.9	0.952	6.2 ± 5.3	5.3 ± 4.8	0.056	7.0 ± 5.0	7.9 ± 5.7	0.244	9.3 ± 9.9	7.1 ± 6.6	0.018
*Long term HRV, ms*												
ASDNN	50.7 ± 21.8	53.5 ± 21.8	0.174	48.4 ± 15.6	54.9 ± 16.2	0.013	48.2 ± 15.1	49.9 ± 16.3	0.612	44.2 ± 17.8	44.5 ± 18.2	0.871
SDANN	105.4 ± 44.7	113.3 ± 39.5	0.149	101.4 ± 36.8	109.8 ± 41.1	0.150	98.0 ± 32.4	104.5 ± 28.6	0.094	99.2 ± 28.4	100.4 ± 30.7	0.794
SDNN	119.6 ± 48.0	128.7 ± 42.9	0.117	114.0 ± 36.4	124.2 ± 37.5	0.078	114.0 ± 37.5	118.7 ± 28.9	0.393	110.8 ± 32.3	113.0 ± 33.8	0.643
*Short term HRV*												
SDNN, ms	18.0 (11.2, 26.8)	14.6 (7.4, 22.1)	0.057	20.6 (13.7, 29.2)	16.4 (9.2, 25.4)	0.264	19.0 (10.2, 29.0)	15.5 (10.8, 30.2)	0.927	16.2 (11.6, 23.2)	10.1 (8.1, 16.8)	0.003
Mean RR, ms	788.9 ± 156.2	753.4 ± 152.8	0.027	796.2 ± 150.9	802.0 ± 131.2	0.799	767.2 ± 143.8	806.8 ± 146.7	0.011	738.2 ± 124.9	714.8 ± 122.2	0.146
RMSSD, ms	18.0 (8.0, 24.5)	11.0 (6.0, 19.0)	0.077	18.0 (12.5, 30.0)	17.0 (8.8, 27.3)	0.157	16.0 (8.0, 22.0)	12.5 (9.0, 27.8)	0.686	14.5 (10.0, 22.8)	9.5 (5.5, 13.0)	0.007
PNS index	− 1.2 ± 1.3	− 1.5 ± 1.2	0.008	− 1.1 ± 1.1	− 1.1 ± 1.0	0.977	− 1.2 ± 0.9	− 1.2 ± 1.0	0.374	− 1.3 ± 1.6	− 1.7 ± 1.1	0.025
SNS index	3.0 ± 2.9	3.9 ± 3.6	0.018	2.3 ± 2.4	2.8 ± 2.4	0.447	3.2 ± 3.0	2.8 ± 2.3	0.438	3.6 ± 3.1	4.7 ± 3.1	0.017
Stress index	23.1 ± 12.4	27.3 ± 17.4	0.028	21.9 ± 14.0	26.7 ± 19.2	0.128	23.6 ± 14.4	23.2 ± 10.3	0.825	25.0 ± 13.6	30.5 ± 13.6	0.019
*24 h heart rate, bpm*												
Min. HR	50.1 ± 9.2	48.6 ± 8.2	0.248	49.3 ± 9.8	46.8 ± 7.0	0.058	48.9 ± 9.7	48.7 ± 6.8	0.849	53.4 ± 9.3	51.9 ± 8.5	0.170
Max. HR	124.1 ± 16.8	122.5 ± 17.0	0.495	123.2 ± 15.7	119.4 ± 13.8	0.091	121.7 ± 15.1	120.8 ± 15.1	0.699	128.9 ± 13.7	126.6 ± 13.0	0.160
Avg. HR	80.9 ± 11.2	79.3 ± 11.1	0.207	79.4 ± 9.9	76.1 ± 8.5	0.037	80.5 ± 8.4	77.7 ± 7.8	0.002	84.3 ± 9.8	84.2 ± 9.8	0.910
Day HR	84.3 ± 12.5	84.7 ± 12.5	0.760	83.7 ± 9.9	80.2 ± 11.3	0.068	84.6 ± 8.9	83.0 ± 8.7	0.118	88.2 ± 11.1	88.9 ± 11.3	0.616
Noct. HR	71.6 ± 12.8	69.3 ± 10.2	0.124	71.1 ± 12.2	67.2 ± 8.9	0.050	71.6 ± 9.5	68.9 ± 7.7	0.010	74.8 ± 10.4	74.7 ± 10.3	0.962
Dipping, %	14.8 ± 11.1	17.8 ± 8.8	0.082	15.1 ± 9.5	15.6 ± 9.9	0.805	15.3 ± 6.7	16.8 ± 6.9	0.251	14.9 ± 7.6	15.5 ± 9.2	0.747
Non-dipper, n (%)	8 (23.5)	5 (14.7)	0.375	8 (28.6)	6 (21.4)	0.727	6 (18.8)	4 (12.5)	0.727	10 (30.3)	8 (24.2)	0.774
*Renin–angiotensin–aldosterone system parameters*												
PAC, ng/dL	8.95 (5.37, 12.30)	10.50 (7.62, 12.65)	0.062	7.84 (6.54, 11.40)	6.83 (4.37, 10.20)	0.206	9.71 (6.08, 13.20)	9.92 (6.64, 13.20)	0.948	8.75 (6.44, 11.30)	7.82 (6.76, 11.15)	0.893
PRC, μIU/mL	12.79 (5.93, 22.60)	15.97 (9.92, 33.02)	0.231	12.12 (5.38, 17.55)	11.60 (4.96, 22.83)	0.785	10.05 (4.85, 23.52)	11.85 (5.84, 32.10)	0.075	15.12 (10.17, 26.41)	15.27 (9.09, 25.67)	0.908
*Endothelial function*												
FMD, %	6.18 ± 1.37	6.70 ± 1.09	< 0.001	5.89 ± 1.51	6.55 ± 1.93	0.006	6.05 ± 1.64	6.45 ± 1.18	0.028	6.2 ± 1.2	6.6 ± 0.8	0.003
> 7.1%	6 (18.2)	11 (33.3)	0.125	5 (17.2)	8 (27.6)	0.250	4 (12.5)	9 (28.1)	0.063	3 (9.1)	7 (21.2)	0.125

Numerical variables are presented as the mean ± standard deviation or median (IQR), categorical variables are defined as absolute count and percentage

hsCRP: high-sensitivity C-reactive protein; ASDNN: the mean of the 5 min SDNN calculated over 24 h; SDANN: the standard deviation of the average NN interval calculated over 5 min; SDNN: standard deviation of the NN interval; RMSSD: the square root of the mean of the sum of the squares of differences between adjacent NN intervals; PNS: parasympathetic nervous system; SNS: sympathetic nervous system; min: minimum; max: maximum; avg: average; noct: nocturnal; PAC: plasma aldosterone concentration; PRC: plasma renin concentration; FMD: flow mediated dilatation; 25(OH)D: 25-hydroxyvitamin D

^*^Patients who had 25(OH)D < 30 ng/mL at baseline and achieved 25(OH)D > 30 ng/mL at the end of visit

### ESS and QoL scores

Table 6 outlines patient-reported outcomes in ESS and QoL. Groups 1, 2, and 3 reported significant improvements in the QoL scores, except in the domain of social relationships. However, only group 3 exhibited significant reduction in ESS.

**Table 6:** ESS and quality of life changes before and after treatment

Variables	Group 1 n = 36			Group 2 n = 41			Group 3 n = 39			Group 4 n = 37		
	Before	After	p	Before	After	p	Before	After	p	Before	After	p
ESS	8.9 ± 5.0	8.0 ± 4.8	0.259	8.9 ± 5.7	8.6 ± 5.8	0.722	10.2 ± 4.7	7.6 ± 5.2	0.004	9.4 ± 5.7	8.6 ± 4.8	0.254
WHOQoL												
Quality of Life	3.49 ± 0.77	3.97 ± 0.69	< 0.001	3.39 ± 0.70	3.78 ± 0.57	0.002	3.40 ± 0.81	3.88 ± 0.69	0.004	3.61 ± 0.72	3.73 ± 0.80	0.347
General Health	2.97 ± 0.87	3.24 ± 0.72	0.048	3.02 ± 0.69	3.34 ± 0.83	0.008	2.95 ± 0.64	3.60 ± 0.87	< 0.001	3.11 ± 0.97	3.27 ± 0.96	0.245
Domain 1	12.2 ± 1.7	13.2 ± 1.6	0.003	12.5 ± 1.7	13.1 ± 2.0	0.012	12.7 ± 1.6	13.4 ± 2.2	0.014	12.8 ± 1.7	13.2 ± 1.4	0.252
Domain 2	12.8 ± 1.9	13.4 ± 1.8	0.033	12.5 ± 1.6	13.5 ± 1.9	< 0.001	13.1 ± 2.1	13.7 ± 2.4	0.075	13.1 ± 1.7	13.3 ± 1.9	0.497
Domain 3	14.4 ± 2.5	15.1 ± 2.8	0.202	13.9 ± 2.8	14.1 ± 2.6	0.751	13.9 ± 2.9	14.6 ± 3.5	0.217	14.5 ± 3.4	14.5 ± 2.6	1.0
Domain 4	14.1 ± 2.2	14.6 ± 1.6	0.076	13.7 ± 2.1	14.5 ± 2.1	0.007	14.1 ± 1.9	15.0 ± 2.9	0.032	14.6 ± 1.8	15.0 ± 1.7	0.044

Numerical variables are presented as the mean ± standard deviation

ESS: Epworth Sleepiness Scale; WHOQoL: World Health Organization Quality of Life; Domain 1: physical health; Domain 2: psychological health; Domain 3: social relationships; Domain 4: environmental health

### Adverse events

Three patients in group 1 developed genitourinary infection during the study period, which subsequently resolved with treatment. There were more patients in group 3 who developed other infections as shown in Supp Table 5. None of the patients who received Dapagliflozin developed ketoacidosis.

## Discussion

This study demonstrated three pertinent findings. Firstly, untreated OSA has deleterious effects on the cardiovascular system over time, highlighting the progressive nature of metabolic dysfunction in OSA patients. Secondly, the combination of SGLT2i and vitamin D3 provide a synergistic benefit in metabolic improvement and prevention of cardiac autonomic function deterioration, particularly in females. Thirdly, the use of this combination therapy led to significant improvements in both sleepiness symptoms as well as QoL. To the best of our knowledge, this is the first study evaluating the synergistic effect of combining SGLT2i and vitamin D3 in cardio-metabolic parameters, sleepiness symptoms, and QoL in a group of OSA patients with high cardiovascular risks.

In untreated OSA patients, cardio-metabolic parameters often continue to worsen due to insulin resistance, intermittent hypoxia, and sympathetic overactivity [36]. Despite an improvement in hsCRP in the control arm, which may reflect the natural variation rather than a true anti-inflammatory effect, the persistent autonomic dysregulation and cardiovascular stress explain the lack of improvement in metabolic parameters and deterioration in HRV over time, highlighting the progressive impact of untreated OSA on autonomic and metabolic health.

The extra-glycemic benefits of SGLT-2i have been well-demonstrated. Apart from lowering blood glucose with minimal adverse effects of hypoglycemia, SGLT2i led to reductions in body weight, visceral fat, and BP [37]. Nevertheless, the lack of impact on cardiac autonomic function as demonstrated in this study suggests that improving metabolic factors alone may not be adequate to protect against cardiac autonomic dysregulation in OSA. In fact, SGLT2i alone could potentially worsen HRV due to volume depletion and subsequent RAAS activation [38], which may lead to increases in SNS activity and worsened HRV. This is demonstrated by an increment in PRC and PAC only in the SGLT2i arm, despite not achieving statistically significant differences.

Although vitamin D3 led to some improvements in metabolic parameters, it was not as robust as SGLT2i treatment. Existing meta-analyses demonstrated contradicting results on the effect of vitamin D3 supplementation on BP, which could be contributed by the various doses and short duration of treatment [39–41]. In the current study, vitamin D3 led to improvement in BP among those who achieved vitamin D sufficiency at the end of follow up. Furthermore, vitamin D3 use was demonstrated to exert protective effect on cardiac autonomic function by preventing the deterioration in HRV expected in untreated OSA, which could be attributed to the direct effects of vitamin D3 on the physiology of the cardiomyocytes [42].

In addition to achieving comparable metabolic improvements to the SGLT2i monotherapy group, the combination of SGLT2i and vitamin D3 effectively prevented the deterioration of cardiac autonomic function, which was otherwise observed in the control group and the SGLT2i monotherapy arm. This finding suggests a synergistic effect of the combined treatment on both metabolic and cardiovascular autonomic function. The protective role of vitamin D in reducing oxidative stress and preserving cardiac autonomic function [43, 44], together with the ability of SGLT2i to improve glycemic control, metabolic parameters, and reduce fluid overload, may work in concert to mitigate cardio-metabolic dysfunction seen in untreated OSA. Moreover, only the combination treatment resulted in significant reduction in nocturnal heart rate, along with a decrease in the 24 h average heart rate. Elevated nocturnal heart rate, often referred to as a “non-dipper” profile, has been associated with increased risk of cerebrovascular and cardiovascular diseases, as well as subclinical inflammation [45–47]. The lack of significant results when comparing non-dippers to dippers following treatment may be attributed to the fact that most patients were already classified as dippers at baseline. With fewer non-dippers to observe changes in response to treatment, the statistical power to detect significant differences between the groups was reduced. This baseline distribution may have limited the ability to demonstrate a meaningful shift in dipping status as a treatment effect.

This study demonstrated that the female patients who received the combination treatment of SGLT-2i and vitamin D3 exhibited greater improvements in both metabolic and cardiovascular parameters compared to their male counterparts. These findings suggest potential gender-specific differences in response to the combination therapy. Estrogen is known to exert cardioprotective effects and play a key role in metabolic regulation [48, 49], which may have contributed to the more pronounced benefits observed in the female patients. Additionally, gender differences in vitamin D metabolism and gene expression may have also played a role in modulating these outcomes [50].

SGLT2i monotherapy has been demonstrated to improve QoL among patients with heart failure [51] and type 2 diabetes [52], but presents conflicting findings regarding the impact on ESS in OSA patients with type 2 diabetes [53, 54]. Furthermore, the sole study evaluating the effects of vitamin D supplementation on ESS in OSA patients reported no significant improvement in sleepiness symptom following treatment [55]. There is no data on the influence of vitamin D supplementation on QoL among OSA patients. Similarly, in other disease entities, this area remains under-researched [56]. Our study demonstrates that, while all three intervention arms showed improvements in QoL scores, only the combination therapy group achieved significant improvements in both QoL and ESS scores. While no statistically significant differences were observed between the groups at baseline, the slightly higher starting ESS in the combination arm could have provided more room for improvement in the symptoms, contributing to the observed reduction. Nonetheless, the consistent improvements observed across multiple outcomes suggest that the combination treatment likely offered meaningful therapeutic benefits. This highlights the potential of this treatment combination as a more comprehensive approach to managing hypertensive OSA with metabolic dysfunction, addressing multiple aspects in cardiac health, metabolic function, sleepiness symptoms, and QoL.

Our study is not without limitations. Firstly, we included only hypertensive OSA patients who were obese, which may limit the generalizability of the findings to other groups, such as normotensive OSA patients, or those with normal BMI. Besides, the 16-week study period may not have been long enough to capture the full range of physiological and metabolic changes associated with the interventions, hence limiting the ability to assess the long-term durability of the observed effects, particularly cardiovascular benefits, which may evolve over a longer duration. Moreover, despite controlling for some variables, other unmeasured factors such as sleep quality and lifestyle changes could have influenced the outcomes, potentially confounding the effects of the interventions. In addition, individual responses to the interventions could vary, which may mask specific benefits that could have been more apparent with each intervention.

Nevertheless, this is the first study investigating the combination of SGLT2i and vitamin D3 in improving cardio-metabolic parameters, daytime somnolence, and QoL in OSA patients with high cardiovascular risks, contributing to the growing body of evidence examining the complex interactions in these aspects. The randomized controlled 4-arm study design helped minimize bias and enabled the comparison of multiple treatment strategies, offering valuable insights into the relative effectiveness of each approach. This facilitates a more exquisite understanding of how different interventions influence various clinical outcomes in patients with OSA. Additionally, by evaluating multiple aspects, including metabolic and cardiovascular parameters alongside to sleepiness symptoms and QoL, this study provides a comprehensive overview of the effects of each treatment. The investigation into the HRV offered valuable insights into cardiac autonomic function, a crucial indicator of cardiovascular risk, particularly in patients with OSA. While our study was not designed to directly investigate the underlying mechanisms of the improvement observed with the combination therapy for heart disease, kidney failure, and autonomic function, the study findings suggest a potential role for this combination therapy in helping to mitigate key risk factors associated with cardiovascular and renal complications. Future research should focus on elucidating the precise mechanistic pathways involved, potentially paving the way for more targeted and effective treatment strategies.

## Conclusions

This study, the first to evaluate multiple outcomes in relation to metabolic and cardiovascular health alongside patient-reported outcomes relevant to hypertensive obese OSA patients, highlights the beneficial synergistic effects of combining vitamin D3 and SGLT2i. This combination not only improves key metabolic parameters but also helps mitigate the deterioration of cardiac autonomic function, while reducing daytime somnolence and enhancing QoL, particularly in female patients. These findings underscore the potential of this combination therapy in addressing the metabolic and cardiovascular challenges, ultimately improving overall health outcomes in these patients. Further long-term studies are essential to confirm the durability of these benefits and to better understand the underlying mechanisms driving the observed improvements.

## Supplementary Information

Supplementary Material 1

## Data Availability

The data that supports the findings of this study are available from the corresponding author upon reasonable request.

## Abbreviations

OSA: Obstructive sleep apnea
QoL: Quality of life
CPAP: Continuous positive airway pressure
RAAS: Renin–angiotensin–aldosterone system
VDD: Vitamin D deficiency
SGLT2i: Sodium glucose transporter-2 inhibitor
BP: Blood pressure
BMI: Body mass index
WHO: World Health Organization
AHI: Apnoea hypopnea index
25(OH)D: 25-Hydroxyvitamin D
MASLD: Metabolic-associated steatotic liver disease
hsCRP: High-sensitivity C-reactive protein
HRV: Heart rate variability
SDNN: Standard deviation of the NN interval
ASDNN: Mean of the 5-min SDNN calculated over 24 h
SDANN: The standard deviation of the average NN interval calculated over 5 min
RMSSD: Square root of the mean of the sum of the squares of differences between adjacent NN intervals
PNS: Parasympathetic nervous system
SNS: Sympathetic nervous system
PAC: Plasma aldosterone concentration
PRC: Plasma renin concentration
FMD: Flow-mediated dilatation
ESS: Epworth Sleepiness Scale
