# Supplementary material for: Unveiling the benefits of Vitamin D3 with SGLT-2 inhibitors for hypertensive obese obstructive sleep apnea patients
Source: J Transl Med. 2025 Mar 7;23:296. doi: 10.1186/s12967-025-06312-w (PMC11889775; doi:10.1186/s12967-025-06312-w)
Supplement: Supplementary file 1 — Supplementary Material 1 [file 12967_2025_6312_MOESM1_ESM.zip › Supp Fig.docx]

Visit 2 (8-week after visit 1): Measurements for anthropometry, steatotic liver disease, endothelial function, HRV, blood tests for metabolic and cardiovascular parameters, vitamin D3 dose titration

Follow-up phone call at 1-week and 4-week after visit 1

Visit 1: Measurements for anthropometry, steatotic liver disease, endothelial function, HRV, blood tests for metabolic and cardiovascular parameters, ESS, QoL questionnaire, treatment allocation

Control arm

N = 37

Defaulted = 1

Dapagliflozin + Vitamin D3 arm

N = 39

Defaulted = 1

Non-compliant = 1

Vitamin D3 arm

N = 41

Non-compliant = 4

Dapagliflozin arm

N = 36

Defaulted = 2

Non=compliant = 1

Patients fulfilled study criteria

N = 163

Patients suspected of having OSA

N = 797

Visit 3 (16-week after visit 1): Measurements for anthropometry, steatotic liver disease, endothelial function, HRV, blood tests for metabolic and cardiovascular parameters, ESS, QoL questionnaire

Supp Fig Study design
