# Supplementary material for: Unveiling the benefits of Vitamin D3 with SGLT-2 inhibitors for hypertensive obese obstructive sleep apnea patients
Source: J Transl Med. 2025 Mar 7;23:296. doi: 10.1186/s12967-025-06312-w (PMC11889775; doi:10.1186/s12967-025-06312-w)
Supplement: Supplementary file 1 — Supplementary Material 1 [file 12967_2025_6312_MOESM1_ESM.zip › Supp Table 1.docx]

**Supp table 1** Mean ± SD and range of short- and long-term HRV parameters summarized from available cross-sectional studies data (adapted from Nunan et al.(1) and Task Force of the European Society of Cardiology the North American Society of Pacing Electrophysiology(2))

| HRV measures | Normal values/ranges |
| --- | --- |
| *Short term* |  |
| SDNN, ms | 32-93 |
| Mean RR, ms | 785-1160 |
| RMSSD, ms | 19-75 |
| Stress index | 7-12 |
| *Long term* |  |
| SDNN, ms | 141±39 |
| SDANN, ms | 127±35 |

**References**

1. Nunan D, Sandercock GR, Brodie DA. A quantitative systematic review of normal values for short-term heart rate variability in healthy adults. Pacing Clin Electrophysiol. 2010;33(11):1407-17.

2. Heart rate variability: standards of measurement, physiological interpretation and clinical use. Task Force of the European Society of Cardiology and the North American Society of Pacing and Electrophysiology. Circulation. 1996;93(5):1043-65.
