# Supplementary material for: Unveiling the benefits of Vitamin D3 with SGLT-2 inhibitors for hypertensive obese obstructive sleep apnea patients
Source: J Transl Med. 2025 Mar 7;23:296. doi: 10.1186/s12967-025-06312-w (PMC11889775; doi:10.1186/s12967-025-06312-w)
Supplement: Supplementary file 1 — Supplementary Material 1 [file 12967_2025_6312_MOESM1_ESM.zip › Supp table 2.docx]

**Supp table 2** Interpretation of parasympathetic nervous system and sympathetic nervous system zones (from Kubios user guide)

| PNS zone | Interpretation | SNS zone | Interpretation |
| --- | --- | --- | --- |
| <-2 | Very low | >2 | Very high |
| -2 to -1 | Low | 1 to 2 | High |
| -1 to 1 | Normal | -1 to 1 | Normal |
| 1 to 2 | High | -2 to -1 | Low |
| >2 | Very high | <-2 | Very low |
