# Supplementary material for: Unveiling the benefits of Vitamin D3 with SGLT-2 inhibitors for hypertensive obese obstructive sleep apnea patients
Source: J Transl Med. 2025 Mar 7;23:296. doi: 10.1186/s12967-025-06312-w (PMC11889775; doi:10.1186/s12967-025-06312-w)
Supplement: Supplementary file 1 — Supplementary Material 1 [file 12967_2025_6312_MOESM1_ESM.zip › Supp Table 3.docx]

**Supp table 3** Metabolic parameter changes before and after treatment in subgroup analysis*

| Metabolic  Variables | Group 1  n=33 | | | Group 2  n=29 | | | Group 3  n=32 | | | Group 4  n=33 | | |
| --- | --- | --- | --- | --- | --- | --- | --- | --- | --- | --- | --- | --- |
|  | Before | After | p | Before | After | p | Before | After | p | Before | After | p |
| *Anthropometric* | | | | | | | | | | | | |
| Systolic BP, mmHg | 154.4±16.7 | 145.3±15.7 | 0.008 | 150.2±14.7 | 140.9±15.5 | 0.004 | 151.9±14.9 | 144.1±17.9 | 0.009 | 156.2±20.9 | 149.4±20.9 | 0.006 |
| Diastolic BP, mmHg | 98.5±11.5 | 93.9±12.6 | 0.028 | 92.9±10.2 | 88.9±11.3 | 0.020 | 97.0±13.9 | 91.9±11.2 | 0.017 | 100.3±14.8 | 96.5±14.5 | 0.029 |
| Pulse rate, bpm | 76.5±14.5 | 75.0±14.7 | 0.280 | 74.2±13.5 | 70.4±11.8 | 0.126 | 76.5±12.4 | 73.3±10.9 | 0.118 | 81.9±13.9 | 77.9±14.6 | 0.052 |
| BMI, kg/m^2^ | 42.5±7.5 | 41.1±6.7 | 0.001 | 40.7±6.9 | 40.7±7.2 | 0.838 | 41.9±7.0 | 40.6±6.5 | <0.001 | 40.2±7.9 | 40.1±8.1 | 0.630 |
| Neck circ., cm | 43.3±4.8 | 42.2±5.1 | <0.001 | 42.6±4.4 | 42.3±4.4 | 0.384 | 44.6±4.0 | 43.6±4.3 | <0.001 | 43.1±4.5 | 42.9±4.7 | 0.367 |
| Waist circ., cm | 122.5±15.1 | 118.5±13.7 | <0.001 | 118.9±11.9 | 117.9±13.0 | 0.176 | 123.1±13.0 | 119.1±13.1 | <0.001 | 117.9±16.5 | 117.5±16.5 | 0.475 |
| *Biochemistry* | | | | | | | | | | | | |
| Uric acid, mmol/L | 426.1±86.5 | 349.1±91.8 | <0.001 | 421.5±70.7 | 392.6±86.1 | 0.041 | 443.9±86.9 | 362.1±80.9 | <0.001 | 416.1±86.8 | 418.4±87.2 | 0.825 |
| HbA1c, % | 6.4 (5.9, 6.8) | 6.1 (5.8, 6.5) | 0.005 | 6.4 (6.0, 6.9) | 6.3 (6.0, 7.2) | 0.990 | 6.7 (5.9, 7.7) | 6.1 (5.7, 6.7) | <0.001 | 6.0 (5.8, 7.0) | 6.1 (5.7, 6.7) | 0.337 |
| Lipid, mmol/L  Total  LDL-C  HDL-C  Triglyceride | 4.69±1.35  2.70±1.12  1.29±0.23  1.39 (1.08, 2.14) | 4.35±1.08  2.52±0.96  1.17±0.21  1.30 (1.07, 2.26) | 0.013  0.132  <0.001  0.186 | 4.85±0.91  2.79±0.84  1.34±0.26  1.61 (1.10, 2.41) | 4.35±1.02  2.47±0.86  1.26±0.29  1.33 (1.08, 1.92) | <0.001  0.015  0.051  0.050 | 4.68±1.08  2.71±0.81  1.23±0.24  1.62 (1.10, 2.05) | 4.45±1.06  2.58±0.80  1.17±0.24  1.54 (1.04, 2.00) | 0.184  0.302  0.093  0.089 | 4.68±0.94  2.71±0.80  1.26±0.24  1.65 (1.06, 2.24) | 4.59±0.97  2.68±0.86  1.21±0.22  1.68 (1.27, 2.24) | 0.473  0.840  0.152  0.580 |
| *Ultrasound* | | | | | | | | | | | | |
| MASLD, n (%)  Normal  Grade 1  Grade 2  Grade 3 | 7 (21.2)  5 (15.2)  19 (57.6)  2 (6.1) | 9 (27.3)  4 (12.1)  18 (54.5)  2 (6.1) | 0.50 | 2 (6.9)  8 (27.6)  17 (58.6)  2 (6.9) | 2 (6.9)  8 (27.6)  17 (58.6)  2 (6.9) | 1.0 | 2 (6.5)  6 (19.4)  19 (61.3)  4 (12.9) | 4 (12.9)  4 (12.9)  19 (61.3)  4 (12.9) | 0.50 | 3 (9.1)  6 (18.2)  21 (63.6)  3 (9.1) | 3 (9.1)  6 (18.2)  21 (63.6)  3 (9.1) | 1.0 |

*Patients with serum 25(OH)D <30 ng/dL at baseline and achieved 25(OH)D >30ng/dL at the end of treatment

Numerical variables are presented as the mean ± standard deviation or median (IQR) while categorical variables are defined as absolute count and percentage

BP: blood pressure; BMI: body mass index; circ: circumference; MASLD: metabolic-associated steatotic liver disease
