# Supplementary material for: Unveiling the benefits of Vitamin D3 with SGLT-2 inhibitors for hypertensive obese obstructive sleep apnea patients
Source: J Transl Med. 2025 Mar 7;23:296. doi: 10.1186/s12967-025-06312-w (PMC11889775; doi:10.1186/s12967-025-06312-w)
Supplement: Supplementary file 1 — Supplementary Material 1 [file 12967_2025_6312_MOESM1_ESM.zip › Supp Table 4.docx]

**Supp Table 4** Comparison of metabolic and cardiovascular parameters before and after treatment between genders for group 3 in subgroup analysis*

| Variables | Male  n=19 | |  | Female  n=13 | |  |
| --- | --- | --- | --- | --- | --- | --- |
|  | Before | After | p | Before | After | p |
| *Anthropometric* |  |  |  |  |  |  |
| Systolic BP, mmHg | 152.5±15.0 | 147.1±19.0 | 0.110 | 150.9±15.4 | 139.7±15.8 | 0.043 |
| Diastolic BP, mmHg | 97.5±13.6 | 93.6±10.3 | 0.178 | 96.3±14.7 | 89.4±12.4 | 0.035 |
| Pulse rate, bpm | 78.4±12.6 | 74.3±11.2 | 0.116 | 73.7±12.1 | 72.0±10.7 | 0.609 |
| BMI, kg/m^2^ | 40.4±8.1 | 39.3±7.4 | 0.011 | 44.1±4.6 | 42.6±4.5 | 0.002 |
| Neck circ., cm | 46.5±3.6 | 45.6±3.8 | 0.018 | 42.0±3.0 | 40.6±3.1 | <0.001 |
| Waist circ., cm | 123.8±14.2 | 119.8±13.2 | 0.001 | 122.1±11.4 | 118.0±13.4 | 0.001 |
|  |  |  |  |  |  |  |
| *Biochemistry* |  |  |  |  |  |  |
| Uric acid, mmol/L | 454.4±74.1 | 369.1±79.6 | <0.001 | 428.6±104.0 | 351.8±84.7 | 0.002 |
| HbA1c, % | 6.4 (6.0, 7.0) | 6.9 (5.7, 6.6) | 0.001 | 7.5 (5.9, 7.9) | 6.6 (5.9, 7.5) | 0.017 |
| Total cholesterol, mmol/L | 4.52±0.84 | 4.25±1.04 | 0.154 | 4.91±1.36 | 4.74±1.06 | 0.614 |
| LDL-C, mmol/L | 2.71±0.68 | 2.43±0.78 | 0.065 | 2.73±1.0 | 2.80±0.83 | 0.763 |
| HDL-C, mmol/L | 1.16±0.19 | 1.11±0.23 | 0.248 | 1.34±0.27 | 1.27±0.25 | 0.244 |
| Triglyceride, mmol/L | 1.33 (0.96, 1.92) | 1.47 (1.0, 2.0) | 0.732 | 1.78 (1.40, 2.32) | 1.61 (1.09, 2.04) | 0.028 |
| hsCRP | 5.3±4.5 | 5.9±4.5 | 0.394 | 9.5±4.6 | 10.7±6.2 | 0.433 |
|  |  |  |  |  |  |  |
| *Long term HRV, ms* |  |  |  |  |  |  |
| ASDNN | 52.9±15.7 | 50.3±12.9 | 0.426 | 41.3±11.6 | 49.3±20.9 | 0.228 |
| SDANN | 113.6±32.1 | 113.0±31.7 | 0.908 | 75.1±14.2 | 92.0±18.0 | 0.002 |
| SDNN | 129.2±37.8 | 124.2±29.9 | 0.483 | 91.8±24.2 | 110.7±26.4 | 0.025 |
|  |  |  |  |  |  |  |
| *Short term HRV* |  |  |  |  |  |  |
| SDNN, ms | 16.8 (12.3, 29.0) | 17.7 (11.1, 27.9) | 0.279 | 20.4 (7.6, 29.5) | 13.4 (8.6, 33.0) | 0.333 |
| Mean RR, ms | 782.3±180.7 | 812.5±169.2 | 0.218 | 750.9±94.4 | 800.7±125.0 | 0.013 |
| RMSSD, ms | 17.0 (9.0, 27.0) | 12.5 (8.8, 21.3) | 0.346 | 15.5 (7.3, 21.8) | 12.0 (9.3, 38.3) | 0.083 |
| PNS index | -1.24±1.22 | -1.28±0.99 | 0.880 | -1.53±0.62 | -1.14±0.92 | 0.021 |
| SNS index | 3.14±3.18 | 3.04±1.93 | 0.868 | 3.26±2.88 | 2.60±2.71 | 0.286 |
| Stress index | 23.3±14.5 | 23.7±7.9 | 0.897 | 24.1±14.9 | 22.5±13.1 | 0.641 |
|  |  |  |  |  |  |  |
| *24-hour heart rate, bpm* |  |  |  |  |  |  |
| Minimum HR | 46.8±9.3 | 47.0±7.4 | 0.917 | 52.0±9.7 | 51.2±5.2 | 0.682 |
| Maximum HR | 122.8±17.8 | 120.3±14.0 | 0.456 | 120.1±10.3 | 121.5±17.1 | 0.695 |
| Average HR | 80.9±10.2 | 77.4±8.4 | 0.157 | 82.2±6.3 | 78.0±6.5 | <0.001 |
| Day HR | 83.9±10.3 | 82.7±9.7 | 0.499 | 85.6±6.9 | 83.3±7.6 | 0.042 |
| Nocturnal HR | 68.0±10.1 | 67.2±8.3 | 0.636 | 76.3±6.3 | 70.9±6.6 | <0.001 |
|  |  |  |  |  |  |  |
| Dipping, % | 18.9±6.4 | 18.4±8.1 | 0.754 | 10.8±3.6 | 14.7±4.3 | 0.013 |

*Patients who had serum 25(OH)D <30 ng/mL at baseline and achieved 25(OH)D >30 ng/mL at the end of visit

Numerical variables are presented as the mean ± standard deviation or median (IQR)

BP: blood pressure; BMI: body mass index; circ: circumference; hsCRP: high-sensitivity C-reactive protein; ASDNN: the mean of the 5-minute SDNN calculated over 24 hours; SDANN: the standard deviation of the average NN interval calculated over 5 minutes; SDNN: standard deviation of the NN interval; RMSSD: the square root of the mean of the sum of the squares of differences between adjacent NN intervals; PNS: parasympathetic nervous system; SNS: sympathetic nervous system; HR: heart rate; 25(OH)D: 25-hydroxyvitamin D
