# Supplementary material for: Unveiling the benefits of Vitamin D3 with SGLT-2 inhibitors for hypertensive obese obstructive sleep apnea patients
Source: J Transl Med. 2025 Mar 7;23:296. doi: 10.1186/s12967-025-06312-w (PMC11889775; doi:10.1186/s12967-025-06312-w)
Supplement: Supplementary file 1 — Supplementary Material 1 [file 12967_2025_6312_MOESM1_ESM.zip › Supp Table 5.docx]

**Supp table 5** Adverse events reported among intervention groups

| Adverse events | Group 1 | Group 2 | Group 3 | Group 4 | p |
| --- | --- | --- | --- | --- | --- |
| Genitourinary infection | 3 (8.3) | 0 (0) | 0 (0) | 0 (0) | 0.019 |
| Upper respiratory tract infection | 2 (5.6) | 5 (12.2) | 2 (5.1) | 6 (16.2) | 0.298 |
| Other infections | 2 (5.6) | 1 (2.4) | 6 (15.4) | 0 (0) | 0.023 |
| Diarrhoea | 0 (0) | 0 (0) | 1 (2.6) | 1 (2.7) | 0.561 |

Other infections: HFMD, herpes, carbuncle, fungal infection, cellulitis, thenar abscess
